# Supplementary material for: Selection of Conditions in PVB Polymer Dissolution Process for Laminated Glass Recycling Applications
Source: Polymers (Basel). 2022 Nov 24;14(23):5119. doi: 10.3390/polym14235119 (PMC9737715; doi:10.3390/polym14235119)
Supplement: Supplementary file 1 [file polymers-14-05119-s001.zip › polymers-2045213-supplementary.pdf]

Table S1. PVB weight percent in IPA as a function of the dissolution time, *t* at *T* = 298.15 K, the stirrer speed of 500 rpm, and polymer size 30.0 × 30.0 × 0.82 mm.

t/h	*wt*% PVB	DISS. PVB/%	*t*/h	*wt*% PVB	DISS. PVB/%
Initial volume of 2-propanol, V_{IPA} = 30 ml					
0	0	0	3.20	1.42	48
0.30	0.20	7	4.00	1.67	57
0.60	0.20	7	4.40	1.90	65
1.20	0.53	18	6.40	2.25	77
2.00	0.64	22	8.40	2.69	92
2.40	0.90	31	10.00	2.93	100
Initial volume of 2-propanol, V_{IPA} = 15 ml					
0	0	0	3.20	3.52	56
0.30	0.76	12	4.00	4.10	66
0.60	0.76	12	4.40	4.34	70
1.20	1.67	27	6.40	5.05	81
2.00	2.13	34	8.40	5.95	95
2.40	2.93	47	10.00	6.24	100
Initial volume of 2-propanol, V_{IPA} = 10 ml					
0	0	0	3.20	5.16	54
0.30	0.76	8	4.00	5.88	62
0.60	1.44	15	4.40	6.47	68
1.20	2.24	24	6.40	8.52	90
2.00	3.28	35	8.40	9.25	97
2.40	4.34	46	10.00	9.49	100

wt% PVB is the PVB weight percent in solution, DISS. PVB/% is the precent of dissolved PVB. Standard uncertainties are $u(t)$ = 0.03 h, $u(T)$ = 0.05 K, $u(p)$ = 2 kPa, u(*wt*% PVB) = 0.05, u(Dissolved PVB) = 5 %.

Table S2. PVB weight percent in IPA as a function of the dissolution time, *t* at *T* = 298.15 K, the stirrer speed of 500 rpm, and polymer size 10.0 × 10.0 × 0.82 mm.

t/h	*wt*% PVB	DISS. PVB/%	*t*/h	*wt*% PVB	DISS. PVB/%
Initial volume of 2-propanol, V_{IPA} = 10 ml					
0.00	0.00	0	4.00	0.87	79
0.50	0.20	18	5.50	1.10	100
2.00	0.42	38	7.00	1.10	100
3.00	0.76	69			

wt% PVB is the PVB weight percent in solution, DISS. PVB/% is the precent dissolved PVB. Standard uncertainties are $u(t)$ = 0.03 h, $u(T)$ = 0.05 K, $u(p)$ = 2 kPa, u(*wt*% PVB) = 0.05, u(Dissolved PVB) = 5 %.

Table S3. PVB weight percent in IPA as a function of the dissolution time, *t*, the initial volume of 2-propanol, V_{IPA} = 10 ml, the stirrer speed of 500 rpm, and polymer size 30.0 × 30.0 × 0.82 mm.

t/h	*wt%* PVB	DISS. PVB/%	*t*/h	*wt%* PVB	DISS. PVB/%
		T = 308.15 K			
0.00	0.00	0	3.50	6.19	68
0.50	1.98	22	4.50	7.35	81
1.00	2.67	29	5.50	8.12	89
1.50	3.63	40	6.50	8.95	98
2.00	4.10	45	7.50	9.13	100
2.50	5.05	55	8.50	9.13	100
3.00	5.51	60			
		T = 318.15 K			
0.00	0.00	0	1.60	5.64	61
0.30	1.21	13	2.00	7.07	77
0.60	3.52	38	3.00	9.00	97
1.00	4.10	44	3.30	9.25	100
1.30	5.28	57			

wt% PVB is the PVB weight percent in solution, DISS. PVB/% is the precent dissolved PVB. Standard uncertainties are $u(t)$ = 0.03 h, $u(T)$ = 0.05 K, $u(p)$ = 2 kPa, u(*wt%* PVB) = 0.05, u(Dissolved PVB) = 5 %.

Table S4. PVB weight percent in IPA as a function of the dissolution time, *t*, the initial volume of 2-propanol, V_{IPA} = 15 ml, and polymer size 30.0 × 30.0 × 0.82 mm. Dissolution process with ultrasound.

t/h	*wt%* PVB	DISS. PVB/%	*T*/K	*t*/h	*wt%* PVB	DISS. PVB/%	*T*/K
0.00	0.00	0	296.3	1.25	5.68	93	315.4
0.25	1.85	30	300.2	1.50	6.02	98	317.5
0.50	3.39	55	305.6	2.00	6.13	100	319.3
0.75	3.97	65	308.7	2.25	6.13	100	319.7
1.00	4.89	80	312.2	2.50	6.13	100	320.3

wt% PVB is the PVB weight percent in solution, DISS. PVB/% is the precent dissolved PVB. *T* is the temperature of the ultrasonic bath during the process. Standard uncertainties are $u(t)$ = 0.03 h, $u(T)$ = 0.05 K, $u(p)$ = 2 kPa, u(*wt%* PVB) = 0.05, u(Dissolved PVB) = 5 %.

Table S5. PVB weight percent in { IPA + AcOEt, *V*/*V* = 1/1} as a function of the dissolution time, *t* at *T* = 298.15 K, the stirrer speed of 500 rpm, and polymer size 30.0 × 30.0 × 0.82 mm.

t/h	*wt%* PVB	DISS. PVB/%	*t*/h	*wt%* PVB	DISS. PVB/%
Initial volume of 2-propanol + ethyl acetate, *V*/*V* = 1/1, $V_{IPA+AcOEt}$ = 30 ml					
0.00	0.00	0	1.50	2.63	75
0.25	0.13	4	1.75	2.97	85
0.50	0.59	17	2.00	3.20	91
0.75	1.28	36	2.25	3.52	100
1.00	1.39	40	2.50	3.52	100
1.25	1.86	53			
Initial volume of 2-propanol + ethyl acetate, *V*/*V* = 1/1, $V_{IPA+AcOEt}$ = 15 ml					
0.00	0.00	0	1.50	4.93	73
0.25	0.24	4	1.75	5.36	80
0.50	1.28	19	2.00	5.80	86
0.75	2.41	36	2.25	6.71	100

1.00	2.98	44	2.50	6.71	100
1.25	4.27	64			
Initial volume of 2-propanol + ethyl acetate, *V*/*V* = 1/1, $V_{IPA+AcOEt}$ = 10 ml					
0.00	0.00	0	1.50	5.67	64
0.25	1.17	13	1.75	6.81	77
0.50	2.63	30	2.00	8.74	99
0.75	3.74	42	2.25	8.84	100
1.00	4.60	52	2.50	8.84	100
1.25	4.82	55			

wt% PVB is the PVB weight percent in solution, DISS. PVB/% is the precent of dissolved PVB. Standard uncertainties are *u*(*t*) = 0.03 h, *u*(*T*) = 0.05 K, *u*(*p*) = 2 kPa, *u*(*wt%* PVB) = 0.05, *u*(Dissolved PVB) = 5 %.

Table S6. PVB weight percent in { IPA + AcOEt, *V*/*V* = 1/1} as a function of the dissolution time, *t*, the initial volume of solvent, $V_{IPA+AcOEt}$ = 10 ml, the stirrer speed of 500 rpm, and polymer size 30.0 × 30.0 × 0.82 mm.

t/h	*wt%* PVB	DISS. PVB/%	*t*/h	*wt%* PVB	DISS. PVB/%
T = 308.15 K					
0.00	0.00	0	1.00	6.70	78
0.17	1.66	19	1.25	8.57	100
0.50	3.64	43	1.50	8.57	100
0.75	5.01	58			
T = 318.15 K					
0.00	0.00	0	0.42	5.31	61
0.08	1.14	13	0.50	7.04	80
0.17	2.52	29	0.58	8.77	100
0.25	3.86	44	0.67	8.77	100
0.33	4.97	57			

wt% PVB is the PVB weight percent in solution, DISS. PVB/% is the precent dissolved PVB. Standard uncertainties are *u*(*t*) = 0.03 h, *u*(*T*) = 0.05 K, *u*(*p*) = 2 kPa, *u*(*wt%* PVB) = 0.05, *u*(Dissolved PVB) = 5 %.

Table S7. PVB weight percent in { IPA + AcOEt, *V*/*V* = 1/1} as a function of the dissolution time, *t*, the initial volume of 2-propanol, V_{IPA} = 15 ml, and polymer size 30.0 × 30.0 × 0.82 mm. Dissolution process with ultrasound.

t/h	*wt%* PVB	DISS. PVB/%	*T*/K	*t*/h	*wt%* PVB	DISS. PVB/%	*T*/K
0.00	0.00	0	296.3	1.25	5.35	93	315.4
0.25	1.73	30	300.2	1.50	5.67	98	317.5
0.50	3.19	55	305.6	2.00	5.77	100	319.3
0.75	3.74	65	308.7	2.25	5.77	100	319.7
1.00	4.60	80	312.2	2.50	5.77	100	320.3

wt% PVB is the PVB weight percent in solution, DISS. PVB/% is the precent dissolved PVB. *T* is the temperature of the ultrasonic bath during the process. Standard uncertainties are *u*(*t*) = 0.03 h, *u*(*T*) = 0.05 K, *u*(*p*) = 2 kPa, *u*(*wt%* PVB) = 0.05, *u*(Dissolved PVB) = 5 %.

Table S8. The parameters of eq. 1 for correlation *wt*% PVB in IPA as a function of the dissolution time, *t*.

System	$10^2 \cdot A_1$/h^{-2}	A_2/h	AARD
Solvent: 2-propanol<br>V_{IPA} = **30 ml**<br>PVB size: 30.0 × 30.0 × 0.82 mm<br>T = 298.15 K<br>Stirrer speed: 500 rpm	-1.707	0.4641	0.12
Solvent: 2-propanol<br>V_{IPA} = **15 ml**<br>PVB size: 30.0 × 30.0 × 0.82 mm<br>T = 298.15 K<br>Stirrer speed: 500 rpm	-6.764	1.296	0.079
Solvent: 2-propanol<br>V_{IPA} = **10 ml**<br>PVB size: 30.0 × 30.0 × 0.82 mm<br>T = 298.15 K<br>Stirrer speed: 500 rpm	-9.784	1.927	0.079
Solvent: 2-propanol<br>V_{IPA} = 10 ml<br>PVB size: **10.0 x 10.0 x 0.82 mm**<br>T = 298.15 K<br>Stirrer speed: 500 rpm	-1.956	0.2980	0.090
Solvent: 2-propanol<br>V_{IPA} = 10 ml<br>PVB size: 30.0 × 30.0 × 0.82 mm<br>T = **308.15 K**<br>Stirrer speed: 500 rpm	-0.1557	2.385	0,073
Solvent: 2-propanol<br>V_{IPA} = 10 ml<br>PVB size: 30.0 × 30.0 × 0.82 mm<br>T = **318.15 K**<br>Stirrer speed: 500 rpm	-0.5652	4.667	0.054
Solvent: 2-propanol<br>V_{IPA} = 15 ml<br>PVB size: 30.0 × 30.0 × 0.82 mm<br>Stirrer speed: 500 rpm<br>Ultrasound	-0.3524	2.990	0.12

Table S9. The parameters of eq. 1 for correlation *wt*% PVB in { IPA + AcOEt, *V*/*V* = 1/1} as a function of the dissolution time, *t*.

System	$10^2 \cdot A_1$/h^{-2}	A_2/h	AARD
Solvent: 2-propanol + ethyl acetate<br>V_{IPA} = **30 ml**<br>PVB size: 30.0 × 30.0 × 0.82 mm<br>*T* = 298.15 K<br>Stirrer speed: 500 rpm	-5.043	1.7007	0.32
Solvent: 2-propanol + ethyl acetate<br>V_{IPA} = **15 ml**<br>PVB size: 30.0 × 30.0 × 0.82 mm<br>*T* = 298.15 K<br>Stirrer speed: 500 rpm	-5.723	3.112	0.27
Solvent: 2-propanol + ethyl acetate<br>V_{IPA} = **10 ml**<br>PVB size: 30.0 × 30.0 × 0.82 mm<br>*T* = 298.15 K<br>Stirrer speed: 500 rpm	-53.93	5.141	0.065
Solvent: 2-propanol + ethyl acetate<br>V_{IPA} = 10 ml<br>PVB size: 30.0 × 30.0 × 0.82 mm<br>*T* = **308.15 K**<br>Stirrer speed: 500 rpm	-1.781	6.882	0.070
Solvent: 2-propanol + ethyl acetate<br>V_{IPA} = 10 ml<br>PVB size: 30.0 × 30.0 × 0.82 mm<br>*T* = **318.15 K**<br>Stirrer speed: 500 rpm	-1.779	14.93	0.047
Solvent: 2-propanol + ethyl acetate<br>V_{IPA} = 15 ml<br>PVB size: 30.0 × 30.0 × 0.82 mm<br>Stirrer speed: 500 rpm<br>Ultrasound	-185.8	6.603	0.039

Table S10. The correlation parameters for liquid density of PVB solution in IPA or binary mixture {IPA + AcOEt V/V = 1/1}.

System	$10^6 \cdot b_{11}$/ K^{-1}	$10^3 \cdot b_{12}$	$10^3 \cdot b_{21}$/ K^{-1}	b_{12}	$10^4 \cdot \sigma$/$g \cdot cm^{-3}$
PVB + IPA	-3.123	3.256	-1.050	1.095	4.0
PVB + {IPA + AcOEt V/V = 1/1}	-6.365	2.177	-1.110	1.164	3.6

Table S11. The correlation parameters for liquid dynamic viscosity of PVB solution in IPA or binary mixture {IPA + AcOEt V/V = 1/1}.

System	c_{11}	c_{12}	c_{21}/K	c_{22}/K	σ/mPa·s
PVB + IPA	-0.5680	-7.982	311.5	2733	2.5
PVB + {IPA + AcOEt V/V = 1/1}	-0.6136	-4.071	326.2	1475	7.5

Table S12. The correlation parameters for refractive index of PVB solution in IPA or binary mixture {IPA + AcOEt V/V = 1/1}.

System	$10^4 \cdot D_1$	D_2	$10^4 \cdot \sigma$
PVB + IPA	8.382	1.3752	0.8
PVB + {IPA + AcOEt V/V = 1/1}	9.479	1.3707	1.4

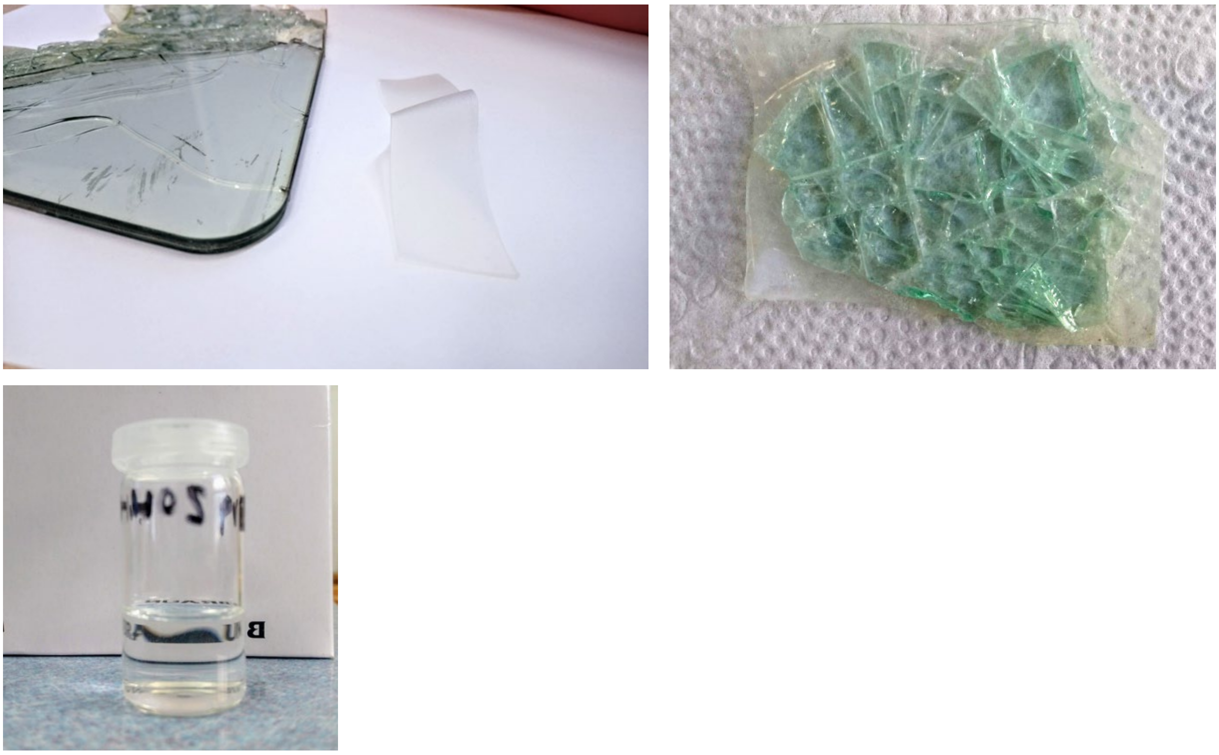

Figure S1. The testing sample of PVB and PVB solution in binary solvent mixtures {2-propanol + ethyl acetate, *V*/*V* = 1/1}.

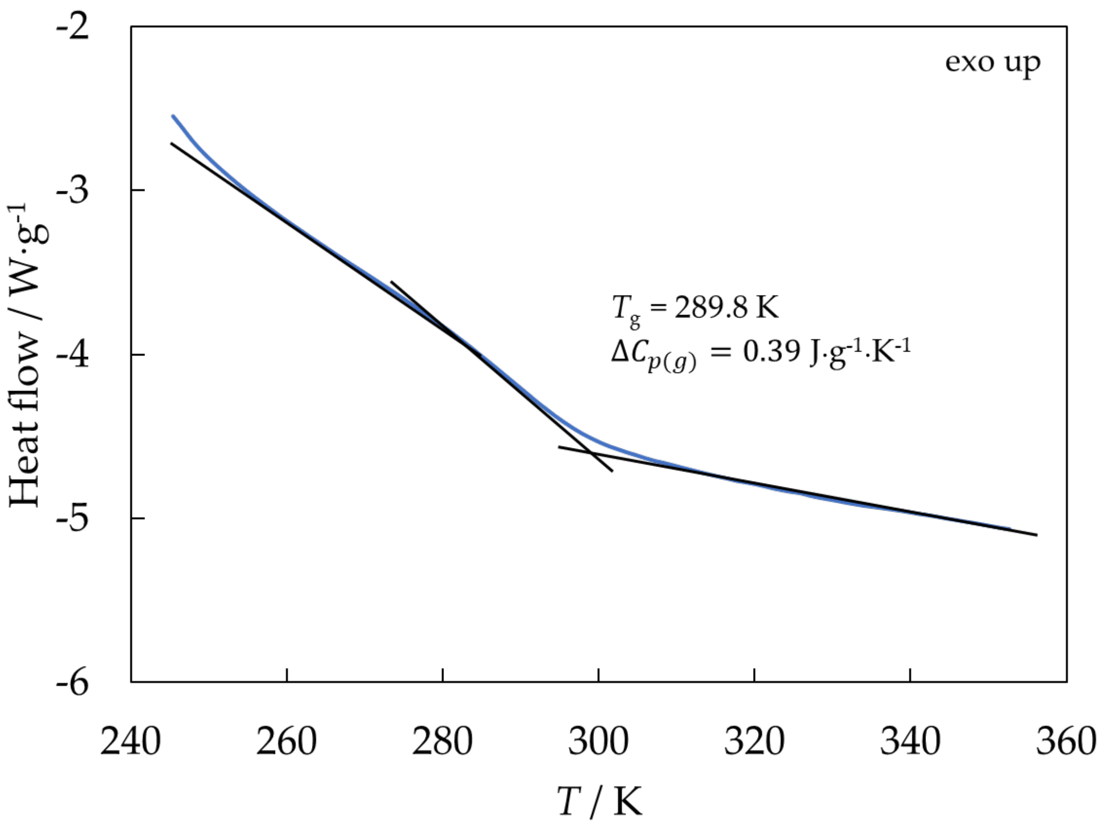

Figure S2. The DSC thermogram of PVB: Glass Transition Temperature, T_g/K; Heat Capacity Change at Glass Transition Temperature, $\Delta C_{p(g)}$/J·g^{-1}·T^{-1}
